# Comparative Analysis of Chlorhexidine Derivatives and Alternative Agents on *Streptococcus mutans* Viability, Biofilm Formation, and Gene Expression

**DOI:** 10.1002/mbo3.70360

**Published:** 2026-07-14

**Authors:** Hamideh Kamalloo, Mohammadreza Moeininejad, Maryam kiani golshoui, Fatemeh Samadyar, Ali Shivaee, Behrooz Sadeghi Kalani

**Affiliations:** ^1^ Department of Microbiology, Science and Research Branch Islamic Azad University Tehran Iran; ^2^ School of Dentistry Tehran University of Medical Sciences Tehran Iran; ^3^ Infectious Diseases and Tropical Medicine Research Center Shahid Beheshti University of Medical Sciences Tehran Iran; ^4^ Department of Microbiology, Faculty of Basic Science, Karaj Branch Islamic Azad University Karaj Karaj Iran; ^5^ Department of Medical Microbiology, School of Medicine Ilam University of Medical Sciences Ilam Iran; ^6^ Student Research Committee Ilam University of Medical Sciences Ilam Iran

**Keywords:** antimicrobial activity, biofilm inhibition, chlorhexidine, *Streptococcus mutans*, virulence gene expression

## Abstract

This study evaluates the antimicrobial and antibiofilm effects of chlorhexidine and related compounds against *S. mutans*, focusing on bacterial viability, biofilm formation, and expression of key biofilm genes.

Eight compounds were tested for their chemical properties, MICs, and interactions via FIC analyses. Time‐kill assays assessed bactericidal activity over 24 h, while cytotoxicity was evaluated in human gingival fibroblasts. Gene expression of biofilm‐related genes was analyzed to determine transcriptional modulation.

Chlorhexidine gluconate and alexidine showed the strongest antimicrobial activity, with MICs of 2 μg/mL against the reference strain and 4 μg/mL for clinical isolates. Time‐kill results demonstrated bacterial reductions from ~7.2 to < 1.0 log CFU/mL in the reference strain but less effect in clinical isolates. Chlorhexidine reduced biofilm biomass by nearly 19% at 48 h, whereas amoxicillin increased biofilm formation by over 12%. Cytotoxicity varied, with proguanil showing high cell viability (90%), while chlorhexidine and alexidine reduced viability to 50‐70%. Gene expression analyzes revealed significant downregulation of *gtf*D and *brp*A by chlorhexidine and analogs, contrasting with mild upregulation by amoxicillin.

These findings highlight chlorhexidine compounds as effective anti‐biofilm agents, emphasizing the need for continued exploration of alternatives and combination therapies to enhance dental caries treatment.

## Graphical abstract

Chlorhexidine gluconate and alexidine potently kill *S. mutans*, disrupt biofilms, and downregulate virulence genes, yet show moderate cytotoxicity. In contrast, sub‐MIC amoxicillin paradoxically promotes biofilm formation, highlighting the need for targeted antibiofilm therapies.

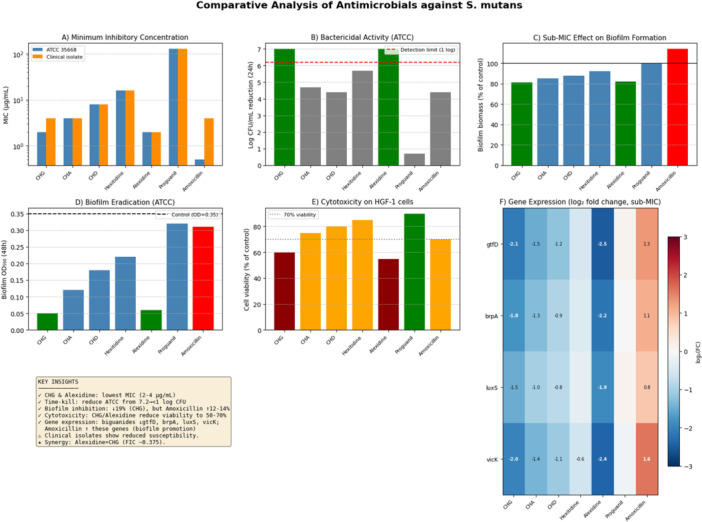

## 1 Introduction

Dental caries remains one of the most widespread chronic diseases globally, imposing substantial economic and public health challenges (Warreth 2023). It is now widely accepted that caries is a polymicrobial disease caused by the entire biofilm community residing on tooth surfaces, rather than by a single bacterial species (Gao 2024). Within this complex consortium, Streptococcus mutans has long been recognized as a key contributor due to its well‐characterized virulence attributes, though it is no longer considered the sole or primary causative agent (Bowen 2018; Hajishengallis et al. 2023; Mira et al. 2017; Kulshrestha and Gupta 2022). *S. mutans* possesses distinct virulence attributes that contribute to its exceptional capacity to colonize and persist within the oral environment. Central to its pathogenicity is the ability to metabolize dietary carbohydrates efficiently, particularly sucrose, via specialized enzymatic pathways. This metabolism drives the synthesis of extracellular polysaccharides by glucosyltransferase enzymes, such as *gtf*D, which are crucial for bacterial adhesion to tooth surfaces and the development of structured, resilient biofilms (Gao 2024).

The formation of biofilms is a critical factor in the pathogenesis of dental caries. These structured microbial communities confer enhanced resistance to antimicrobial agents and host immune responses compared to free‐floating planktonic cells (Gao 2024). Within the cariogenic biofilm, which comprises multiple bacterial species including *S. mutans*, Actinomyces, and non‐mutans streptococci, the synergistic interactions among community members drive disease progression (Mira et al. 2017; Kulshrestha and Gupta 2022; Willems et al. 2016; Nicolas and Lavoie 2011). *S. mutans* expresses genes like *brp*A (Bowen 2018), which regulate biofilm maturation and stability, and *vic*K, involved in two‐component signaling systems that mediate responses to environmental stressors (Hajishengallis et al. 2023). Moreover, quorum sensing genes such as *lux*S coordinate communal behaviors essential for biofilm development and virulence regulation (Mira et al. 2017). The expression of these genes within the biofilm state directly influences the bacterium's ability to sustain localized acidic microenvironments, resist hostile conditions, and maintain its cariogenic potential.

Given the complexity and resilience of *S. mutans* biofilms, monitoring the expression of these critical genes provides valuable insights into the bacterium's adaptive mechanisms under antimicrobial pressure. Understanding how sub‐inhibitory concentrations of compounds affect the transcription of *gtf*D, *brp*A, *lux*S, and *vic*K is vital for identifying therapeutic agents that can disrupt biofilm formation and stability effectively without disturbing the broader oral microbiota. Such targeted approaches hold promise for developing more selective and long‐lasting caries management strategies.

Chlorhexidine has long been the benchmark antimicrobial agent in dental care due to its broad‐spectrum efficacy and robust activity against *S. mutans (*Kulshrestha and Gupta 2022 *)*. Its multifaceted mechanisms of action include disrupting bacterial cell membrane integrity, precipitating cytoplasmic contents, and critically, inhibiting the synthesis of extracellular polysaccharides, which compromises the biofilm structural matrix. This makes chlorhexidine remarkably effective not only at reducing planktonic bacterial populations but also at impairing biofilm development and persistence. Additionally, its substantivity—the ability to bind to oral surfaces and release slowly—ensures sustained antimicrobial effects, which is advantageous for maintaining reduced bacterial loads between applications (Zhang 2021).

Despite these benefits, chlorhexidine use is not without drawbacks (Zhang 2021). Prolonged or excessive application can lead to adverse effects such as tooth staining, taste alteration, mucosal irritation, and disruption of the oral microbiome balance (Deus and Ouanounou 2022; Brookes 2020; McCoy 2008; Wattanawongwan 2025). More concerning is the emerging evidence of reduced susceptibility in certain bacterial strains, which raises the specter of resistance development. These limitations have spurred increased interest in alternative compounds that replicate or enhance chlorhexidine's antimicrobial and anti‐biofilm properties while minimizing side effects (Deus and Ouanounou 2022; Wattanawongwan 2025; Cieplik 2019).

The investigation of chlorhexidine‐like compounds is particularly important because such analogs may offer improved safety profiles, enhanced biofilm penetration, and selective targeting of virulence pathways (Willems et al. 2016). By focusing on how these compounds modulate the expression of key biofilm‐associated genes such as *gtf*D, responsible for glucan‐mediated adhesion; *brp*A, which governs biofilm robustness; *lux*S, a central component of quorum sensing communication; and *vic*K, a sensor kinase that regulates stress response and gene expression within biofilms, researchers can identify novel agents that disrupt biofilm formation more effectively. This gene‐centered approach enables a deeper understanding of how antimicrobial pressure affects bacterial behavior at the molecular level, guiding the rational design of targeted therapies (Nicolas and Lavoie 2011).

Moreover, integrating phenotypic assessments with detailed gene expression analysis offers a comprehensive framework to evaluate candidate compounds' efficacy, antimicrobial potency, and impact on microbial ecology. This is critical for developing therapeutic solutions that not only eradicate pathogens but also preserve the symbiotic balance of the oral microbiome. Ultimately, these efforts aim to overcome the limitations of existing treatments, reduce the risk of resistance development, and deliver effective, safe, and sustainable interventions that enhance clinical management (Bowen 2018).

This study aims to evaluate the antimicrobial and anti‐biofilm efficacy of chlorhexidine and chlorhexidine‐like compounds against Streptococcus mutans. It focuses on assessing their impact on bacterial viability, biofilm formation, and the expression of key biofilm‐associated and virulence genes to identify potential therapeutic agents with improved safety and effectiveness for clinical application.

## 2 Materials and Methods

### 2.1 Bacterial Strains and Culture Conditions

The study utilized two strains of *S. mutans:* the reference strain ATCC 35668 and a clinical isolate obtained from active carious lesions. Both strains were cultured in brain heart infusion broth supplemented with 1% sucrose (BHIS) to support optimal growth. Cultures were maintained at 37°C in a microaerophilic environment (5% CO_2_) to mimic oral cavity conditions. For experimental procedures, overnight cultures were standardized to an optical density of 0.5 at 600 nm, corresponding to approximately 1 × 10^8^ colony forming units per milliliter (Kim 2025).

### 2.2 Antimicrobial Susceptibility Testing

The minimum inhibitory concentrations (MICs) of all test compounds were determined using a standardized broth microdilution method according to CLSI guidelines with modifications for anaerobic bacteria. Two‐fold serial dilutions of each compound were prepared in BHIS broth across 96‐well microtiter plates. The concentration ranges tested were: 0.5–256 μg/mL for chlorhexidine derivatives, 0.25–128 μg/mL for bisbiguanides, 1–512 μg/mL for antimalarial compounds, and 0.125–64 μg/mL for Amoxicillin. Following inoculation with standardized bacterial suspensions and anaerobic incubation, resazurin dye was added as a metabolic indicator to visually determine the MIC endpoints (Wang 2021).

Table 1 compares eight antimicrobial compounds, including chlorhexidine derivatives (gluconate, acetate, dihydrochloride), hexitidine, alexidine, proguanil, and chlorproguanil, highlighting differences in chemical structure, solubility, molecular weight, LogP, charge, stability, and metal ion binding. Chlorhexidine gluconate exhibits the highest water solubility (> 50 mg/mL) and strong metal chelation, while chlorproguanil is poorly soluble (< 0.5 mg/mL) and more hydrophobic. These physicochemical variations directly influence biofilm penetration, antimicrobial efficacy, and formulation stability. For more complete information, please refer to the detailed parameters shown in Table 1.

**Table 1:** Comparative structural and physicochemical properties of selected antimicrobial bisbiguanide and biguanide compounds.

Property	Chlorhexidine gluconate	Chlorhexidine acetate	Chlorhexidine dihydrochloride	Hexitidine	Alexidine	Proguanil	Chlorproguanil
Chemical Structure	Bisbiguanide + gluconate anion	Bisbiguanide + acetate anion	Bisbiguanide + 2HCl	Bisbiguanide + piperidine ring	Bisbiguanide + alkylamine chains	Biguanide + chlorophenyl	Biguanide + dichlorophenyl
Molecular Formula	C_34_H_54_Cl_2_N_10_O_14_	C_22_H_30_Cl_2_N_10_O_2_	C_22_H_30_Cl_2_N_10_·2HCl	C_21_H_44_Cl_2_N_10_O_2_	C_28_H_54_Cl_2_N_10_	C_11_H_16_ClN_5_	C_11_H_15_Cl_2_N_5_
Molecular Weight (g/mol)	897.8	578.4	578.4 (free base) + HCl	503.5	625.7	253.7	288.2
Counterion	Gluconate (C_6_H_11_O_7_^−^)	Acetate (CH_3_COO^−^)	Hydrochloride (Cl^−^)	None (neutral)	None (neutral)	Hydrochloride (Cl^−^)	Hydrochloride (Cl^−^)
Charge at pH 7	Cationic (diquaternary)	Cationic (diquaternary)	Cationic (diquaternary + Cl^−^)	Weakly cationic	Strongly cationic	Weakly basic	Weakly basic
Solubility in Water	High (> 50 mg/mL)	Moderate (~ 20 mg/mL)	Low (~ 5 mg/mL)	Moderate (~10 mg/mL)	High (> 50 mg/mL)	Low (~1 mg/mL)	Very low (< 0.5 mg/mL)
LogP (Partition Coefficient)	~2.1 (hydrophilic)	~2.5	~3.0 (less polar)	~3.2	~1.8 (more polar)	~2.8	~3.5
Key Functional Groups	Two biguanide groups, gluconate (polyol)	Two biguanide groups, acetate	Two biguanide groups, HCl salt	Single biguanide, piperidine ring	Two biguanide groups, ethylhexyl chains	Biguanide, p‐chlorophenyl	Biguanide, 3,4‐dichlorophenyl
pKa	~10.5 (biguanide)	~10.5 (biguanide)	~10.5 (biguanide)	~9.8 (piperidine N)	~11.0 (alkylamine)	~7.5 (biguanide)	~7.2 (biguanide)
Chemical Stability	Stable in solution	Stable (hygroscopic)	Stable (hygroscopic)	Sensitive to oxidation	Very stable	Light‐sensitive	Light‐sensitive
Metal Ion Binding	Strong (chelates Ca^2+^, Mg^2+^)	Moderate	—	—	—	—	—

### 2.3 Synergy Assessment Using Checkerboard Assays

Potential synergistic interactions between compounds were evaluated using checkerboard assays. Various combinations were tested in 96‐well plates with compound concentrations arrayed in two‐dimensional dilutions. After incubation, the fractional inhibitory concentration (FIC) index was calculated for each combination using the formula: (MIC of compound A in combination/MIC of compound A alone) + (MIC of compound B in combination/MIC of compound B alone). Interactions were classified as: synergistic (FIC ≤ 0.5), additive (0.5 < FIC ≤ 1), non‐interactive (1 < FIC ≤ 2), or antagonistic (FIC > 2) (Yuan 2021).

### 2.4 Biofilm Formation and Inhibition Assays

The bacterial biofilm formation and eradication were evaluated using a static microplate assay model. Initially, bacterial suspensions were incubated in BHIS medium to allow biofilm development on polystyrene surfaces for 24 h. To assess biofilm inhibition, mature biofilms were exposed to sub‐minimum inhibitory concentration (sub‐MIC) levels of the test compounds for an additional 24 and 48 h, and the biofilm formation rates were measured relative to untreated controls. Simultaneously, the biofilm eradication potential of the compounds at their MIC doses was evaluated on established biofilms at the same time points (Järvinen et al. 1993).

After treatment, biofilms were gently washed with phosphate‐buffered saline to remove non‐adherent cells, fixed with methanol, and stained with 0.1% crystal violet. The dye bound to the biofilm biomass was subsequently solubilized using 33% acetic acid and quantified by measuring absorbance at 590 nm spectrophotometrically. This method allowed precise quantitation of biofilm biomass to compare the inhibitory and eradication effects of the compounds over time. Overall, this approach enables the distinction between the impact of test agents on both prevention of biofilm formation and disruption of mature biofilms.

### 2.5 Cytotoxicity Evaluation Using MTT Assay

The cytotoxicity of test compounds was evaluated against human gingival fibroblasts (HGF‐1) using the MTT (3‐(4,5‐dimethylthiazol‐2‐yl)‐2,5‐diphenyltetrazolium bromide) assay. Cells were seeded in 96‐well plates and exposed to increasing concentrations of each compound for 24 h. Following treatment, MTT reagent was added and incubated for 4 h to allow formazan crystal formation by viable cells. The crystals were dissolved in dimethyl sulfoxide and the absorbance was measured at 570 nm. Cell viability was expressed as a percentage relative to untreated controls, and IC50 values were calculated using nonlinear regression analysis (Deus and Ouanounou 2022).

### 2.6 RNA Extraction and cDNA Synthesis

For gene expression analysis, bacterial cultures were treated with sub‐inhibitory concentrations of selected compounds for 4 h. Total RNA was extracted using the RNeasy Mini Kit according to the manufacturer's protocol. Briefly, bacterial pellets were lysed using lysozyme and mechanical disruption, followed by RNA binding to silica membranes. After rigorous washing steps, high‐quality RNA was eluted in nuclease‐free water. RNA concentration and purity were verified spectrophotometrically using A260/A280 ratios. cDNA was synthesized from 1 μg of total RNA using the iScript cDNA Synthesis Kit, which combines reverse transcriptase with optimized buffer components for efficient first‐strand synthesis. The reaction mixture included RNA template, random hexamers, dNTPs, reverse transcriptase, and RNase inhibitor, incubated at 25°C for 5 min, 46°C for 20 min, and 95°C for 1 min.

### 2.7 Primer Design for Target Genes

Primers for the target genes (*gtf*D, *brp*A, *lux*S, *vic*K, and *gyr*B) were designed using a targeted in silico strategy. The process began with the retrieval of each gene's complete coding sequence from the NCBI GenBank database. These sequences were then used as input for the Primer3Plus software to design oligonucleotide pairs with optimized parameters, including a unified theoretical annealing temperature of 60°C and amplicon sizes between 100 and 200 bp. The specificity of each primer pair was subsequently validated in silico using NCBI BLASTn to ensure exclusive binding to the intended target gene within the *S. mutans* genome, confirming the predicted amplicon size and absence of significant off‐target homology.

### 2.8 Quantitative Real‐Time PCR Analysis

Gene expression was quantified using SYBR Green‐based real‐time PCR on a StepOnePlus system. Each 20 μL reaction contained: 10 μL SYBR Green Master Mix, 1 μL cDNA template, 1 μL each of forward and reverse primers (10 μM) (Supplementary 1), and 7 μL nuclease‐free water. The thermal cycling protocol included an initial denaturation at 95°C for 10 min, followed by 40 cycles of 95°C for 15 s and 60°C for 1 min. Melting curve analysis was performed to verify amplification specificity. Target genes included virulence and biofilm‐related factors—*gtf*D, *brp*A, *lux*S, and *vic*K. The housekeeping gene *gyr*B served as an internal control. To ensure accurate and reproducible quantification in qPCR, the amplification efficiency for each primer pair was experimentally determined. Relative gene expression was calculated using the 2^^(−ΔΔCt)^ method, comparing treated samples to untreated controls (Brookes 2020).

### 2.9 Statistical Analysis

All experiments were performed with three independent biological replicates, each containing three technical replicates. Data were analyzed using one‐way analysis of variance (ANOVA) followed by Tukey's post‐hoc test for multiple comparisons. Results were considered statistically significant at *p* < 0.05. All statistical analyzes were performed using GraphPad Prism version 9.0 software. Data are presented as mean ± standard deviation unless otherwise specified.

## 3 Results

### 3.1 MIC

MIC values for *S. mutans* ATCC 35668 and clinical isolates are presented in Table 2. CHG and Alexidine showed low MICs of 2 μg/mL against the ATCC strain, indicating strong antimicrobial activity. Clinical isolates required a slightly higher CHG concentration (4 μg/mL), suggesting reduced susceptibility. Amoxicillin had a very low MIC (0.5 μg/mL) against ATCC but a higher MIC (4 μg/mL) for clinical isolates. Proguanil showed very high MICs (> 128 μg/mL) against both strains, indicating poor efficacy. CHA, CHD, Hexitidine, and Alexidine had equal MICs for both strains, reflecting consistent susceptibility.

**Table 2:** Minimum inhibitory concentration (MIC) values of antimicrobial compounds against *S. mutans* ATCC 35668 and clinical isolates.

Compound	ATCC 35668 MIC (μg/mL)	Clinical Isolate MIC (μg/mL)
CHG	2	4
CHA	4	4
CHD	8	8
Hexitidine	16	16
Alexidine	2	2
Proguanil	> 128	> 128
Amoxicillin	0.5	4

### 3.2 Fractional Inhibitory Concentration (FIC) Indices

**Figure 1:**
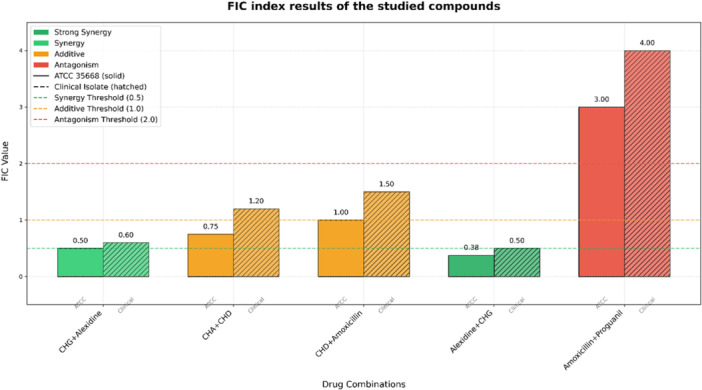
Evaluation of drug interaction outcomes against *S. mutans* strains using fractional inhibitory concentration (FIC) indices.

FIC indices for various drug combinations against both *S. mutans* strains are shown in Figure 1. Alexidine + CHG showed strong synergy (FIC ~ 0.375) against the ATCC strain, whereas Amoxicillin + Proguanil exhibited antagonism (FIC > 3), particularly pronounced in the clinical isolate. Overall, the clinical isolate tended to have higher FIC values than the ATCC strain, indicating reduced susceptibility and less frequent synergistic interactions. Threshold lines in the plot delineate synergy, additivity, and antagonism zones.

### 3.3 Time‐Kill Kinetics

**Figure 2:**
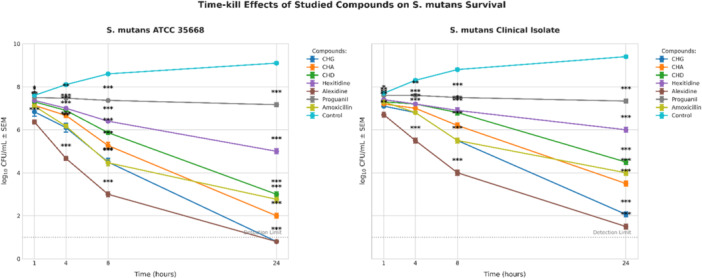
Time‐kill kinetics of antimicrobial compounds against studied *S. mutans* strains.

Time‐kill results over 24 h are shown in Figure 2. CHG and Alexidine were the most effective, reducing ATCC bacterial counts from approximately 7.2 log CFU/mL at 1 h to below the detection limit (1.0 log CFU/mL) at 24 h. Against the clinical isolate, their effects were less pronounced, with bacterial counts lowering only to about 2.0 log CFU/mL, indicating increased tolerance. CHA and CHD reduced ATCC populations by 4–5 logs but only about 3 logs in the clinical isolate after 24 h. Hexitidine showed limited efficacy, particularly against the clinical isolate. Proguanil exhibited no significant antimicrobial activity in either strain. Amoxicillin reduced ATCC counts from ~7.1 to 2.8 log CFU/mL after 24 h, but the clinical isolate showed a more modest reduction to approximately 4.0 log CFU/mL.

### 3.4 Sub‐MIC Effects on Biofilm Formation

**Figure 3:**
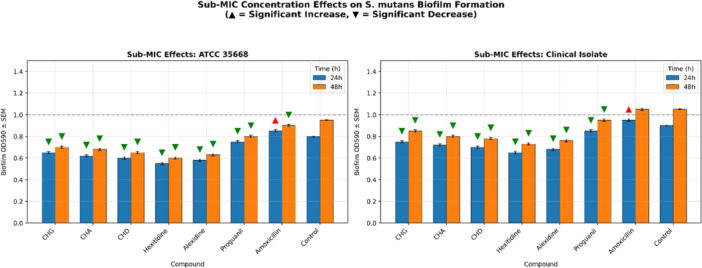
Impact of sub‐inhibitory concentrations (sub‐MIC) of antimicrobial compounds on *S. mutans* biofilm formation.

At sub‐inhibitory concentrations (Figure 3), amoxicillin induced biofilm formation, with biomass increasing by 12.5% in the ATCC strain and 14.3% in the clinical isolate at 48 h. In contrast, biguanides (CHG, CHA, CHD) significantly inhibited biofilm development, with CHG showing the most pronounced reduction (18.8% decrease in ATCC, 19.0% in clinical isolate). Proguanil exhibited no significant anti‐biofilm activity. The clinical isolate consistently showed higher baseline biofilm formation (10%–15% more) and greater resistance to biofilm inhibition. Over time, biofilm biomass increased from 24 to 48 h for all treatments, but biguanides sustained their inhibitory effects while amoxicillin's stimulatory effect amplified.

### 3.5 Biofilm Eradication Capacity

**Figure 4:**
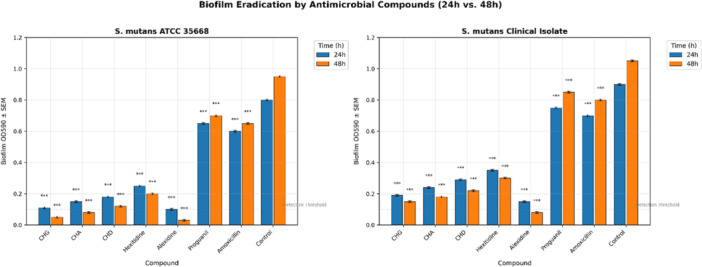
Comparative assessment of antimicrobial compounds to eradicate *S. mutans* biofilm formation.

Figure 4 shows the biofilm eradication capacity of seven compounds. CHG was the most effective, reducing ATCC biofilm OD_590_ to just 0.05 by 48 h. Alexidine showed similarly strong inhibition, although with slightly delayed action. Hexitidine exhibited moderate biofilm reduction. Proguanil and amoxicillin were largely ineffective, maintaining biofilm levels close to untreated controls. The clinical isolate displayed greater inherent biofilm formation and higher residual biofilm after treatment with CHD and hexitidine. All effective compounds showed greater inhibition at 48 versus 24 h (*p* < 0.05).

### 3.6 Cytotoxicity

**Figure 5:**
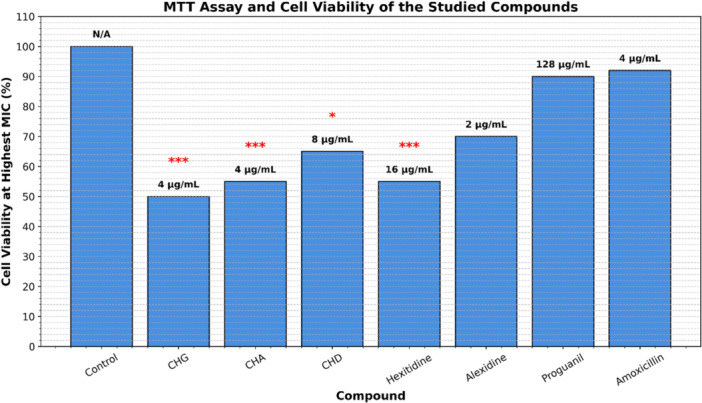
Cytotoxicity of antimicrobial compounds against human gingival fibroblasts (HGF‐1) at their minimum inhibitory concentration (MIC).

MTT assay results (Figure 5) on human gingival fibroblasts (HGF‐1) showed that Proguanil had the least cytotoxicity, with approximately 90% cell viability at its MIC (> 128 μg/mL). CHG and Alexidine displayed the highest cytotoxic effects, reducing viability to around 50%–70% at their respective MICs. Amoxicillin maintained about 70% viability.

### 3.7 Gene Expression

**Figure 6:**
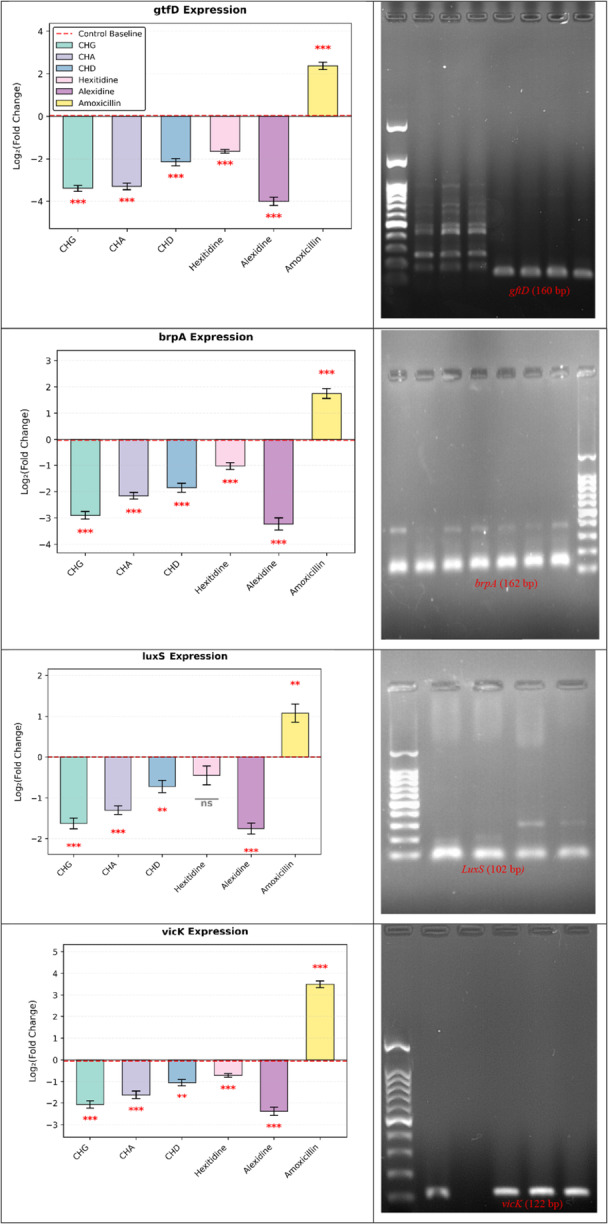
The expression levels of studied genes (*gtfD*, *brpA*, *luxS*, vicK) in S. mutans biofilms following exposure to sub‑MIC concentrations of antimicrobial compounds. On the right side of each graph, a photograph of the corresponding PCR‑gene gel band is provided, including a 100 bp ladder. Graphs present data as the mean (±SD) of three independent experiments, with significance levels denoted as *p < 0.05, **p < 0.01, ***p < 0.001, and ****p < 0.0001 based on statistical analysis using one‑way ANOVA and Tukey's multiple comparisons test compared to the untreated control. The dashed red line indicates the control baseline (mean expression of untreated samples).

The study used real‐time PCR with specifically designed primers to examine target gene expression. All primer pairs showed high amplification efficiency (95%–105%) and strong linear standard curves (*R* ^2^ > 0.99), confirming their suitability for accurate qPCR analysis. The Figure 6 visualizes transcriptional changes under sub‑MICs of antimicrobial compounds, treatment with the biguanide compounds, particularly alexidine and CHG consistently downregulated all four biofilm‐associated genes (*gtfD*, *brpA*, *luxS*, and *vicK*), with alexidine showing the strongest inhibitory effect across most targets (fold changes from −1.8 to −4.0). In contrast, amoxicillin paradoxically upregulated all tested genes, most notably *vicK* and *gtfD* (fold changes +3.2 and +2.5, respectively), consistent with its observed biofilm‑promoting effect. These results indicate that while biguanides suppress virulence gene expression, sub‑MIC amoxicillin may enhance biofilm formation at least in part through transcriptional activation of key regulatory and exopolysaccharide synthesis genes.

## 4 Discussion

The comparative analysis of seven antimicrobial compounds reveals critical insights into their chemical properties, antimicrobial efficacy, and effects on *S. mutans* biofilms. Chlorhexidine derivatives, namely CHG, CHA, and CHD, share bisbiguanide structures with differing counterions and demonstrate strong cationic character and generally high solubility, particularly CHG. These physicochemical traits, well‐documented for their role in facilitating interaction with bacterial membranes (McCoy 2008), underpin their potent antimicrobial properties. Compounds like Hexitidine and Alexidian, which also possess bisbiguanide moieties but vary structurally, exhibit moderate to high solubility and balanced lipophilicity, supporting efficient biofilm penetration and antimicrobial action.

In contrast, Proguanil and Chlorproguanil, although structurally distinct with single biguanide groups and chlorophenyl substitutions, show much lower solubility and higher hydrophobicity. Their chemical instability and light sensitivity suggest limitations in formulation and clinical utility, corresponding with the observed poor antimicrobial efficacy reflected by very high MIC values against both reference and clinical *S. mutans* strains.

The MIC data clearly illustrate that CHG and alexidine are among the most potent antimicrobials tested, exhibiting low MICs against the reference strain. However, the slightly elevated MICs in clinical isolates align with other reports of reduced susceptibility in circulating strains (Wattanawongwan 2025), emphasizing the dynamic challenge of resistance and the necessity of ongoing susceptibility testing. Particularly, amoxicillin's significant increase in MIC from the reference strain to clinical isolates further underscores this emerging resistance that may compromise standard treatments.

The FIC index analysis adds another dimension by revealing interactions between drug combinations. The strong synergy of the chlorhexidine‐alexidine combination against the ATCC strain suggests potential benefits of combined therapy, whereas antagonistic effects observed in combinations such as amoxicillin‐proguanil caution against indiscriminate drug pairing, especially for resistant clinical isolates.

Time‐kill studies confirm the rapid and potent bactericidal effects of CHG and alexidine, significantly reducing bacterial loads within 24 h. This rapid, membrane‑disruptive action is a hallmark of the biguanide class (Wattanawongwan 2025). However, the diminished killing efficacy against clinical isolates indicates the resilience of these strains and challenges the clinical management of persistent infections.

Notably, the investigation into biofilm formation under sub‐MIC conditions exposes critical paradoxes and strain‐specific responses. While biguanide compounds like CHG and alexidine consistently inhibit biofilm biomass, amoxicillin paradoxically enhances it. This finding is consistent with a growing body of literature demonstrating that subinhibitory concentrations of various antibiotics—including β‑lactams, aminoglycosides, and tetracyclines—can promote biofilm formation across multiple bacterial species (Filho et al. 2021; Coelho et al. 2020; Lee and Lee 2015; Šmitran et al. 2018). For instance, sub‑MIC amoxicillin has been shown to induce biofilm matrix production and increase cell surface hydrophobicity in streptococci (Lee and Lee 2015), while similar pro‑biofilm effects have been reported for sub‑MIC erythromycin and clindamycin (Goc 2024). A recent study by Lee and Lee demonstrated that sub‑MIC amoxicillin and penicillin significantly increased biofilm formation in S. mutans, but not in *S. gordonii* or *A. naeslundii*, indicating species‑specific responses within the same oral microbiota (Lee and Lee 2015). Moreover, sub‑MIC amoxicillin has been reported to enhance biofilm formation in Streptococcus suis while simultaneously reducing bacterial virulence in a mouse infection model, suggesting that surviving bacteria shift toward a biofilm‑enclosed persistent state rather than acute virulence (Järvinen et al. 1993). These observations underscore that sub‑therapeutic antibiotic exposure may inadvertently select for more virulent, biofilm‑encased phenotypes, an important consideration for clinical dosing regimens. This finding is particularly insightful when contrasted with other antiseptics; for example, sodium fluoride (NaF) at sub‑MIC levels has been shown to reduce biofilm in a dose‑ and strain‑dependent manner, while sub‑MIC CHX can fail to inhibit biofilms in some strains while effectively reducing it in others. Our results extend these observations by directly comparing multiple biguanides and biguanide‑related compounds, revealing that even within the same chemical class, counterion variation (CHG vs. CHA vs. CHD) can influence sub‑MIC biofilm outcomes, a nuance that has received limited attention in previous reports (Abdelrasoul 2025). The induction of biofilm by amoxicillin, a potentially dangerous bacterial stress response, has significant implications for antibiotic stewardship and dosing strategies, suggesting that sub‑therapeutic exposure may actively promote virulence.

The anti‐biofilm efficacy assessments further underscore CHG and alexidine as leading candidates, with substantial reductions in biofilm mass over time, although clinical isolates show greater inherent resistance. Gene expression profiling provides a mechanistic basis for this: the marked downregulation of pivotal biofilm genes (*gtf*D, *brp*A, *vic*K) by biguanides directly correlates with the observed phenotypic inhibition. This downregulation of key virulence factors is a hallmark of their potent action.

In contrast, the response to sub‑MIC stress can sometimes involve upregulation of regulatory genes, as demonstrated by Jaime Gazola Filho, 2021 (Filho et al. 2021), who found that sub‑MIC CHX and NaF increased expression of the biofilm‑associated vicR and covR. Our gene expression findings align with a larger transcriptomic study by Arbulu and colleagues, who used RNA‑seq to show that sublethal CHX induces major transcriptional remodeling in *S. mutans*, with the response being more pronounced in planktonic cells than in biofilm‑associated cells (Arbulu et al. 2025). Interestingly, that study also observed that biofilm‑associated cells exposed to CHX showed transcriptional responses similar to those induced by amoxicillin, suggesting convergence of stress signaling pathways triggered by different antimicrobial classes (Arbulu et al. 2025). This suggests that the profound suppression of effector genes (*gtfD*, *brpA*) we observed with biguanides may represent a successful overcoming of the bacteria's initial stress response, ultimately shutting down biofilm production. Amoxicillin's mild upregulation of some genes may reflect a different, successful adaptive mechanism under antibiotic stress that ultimately enhances biofilm formation.

Cytotoxicity assays underscore a critical trade‐off between antimicrobial potency and host cell safety.

The significant cytotoxic effects of chlorhexidine gluconate (CHG) on human gingival fibroblasts, which include inducing oxidative stress, cell cycle arrest in the G2/M phase (Coelho et al. 2020), and predominantly necrotic cell death even at concentrations below those used clinically, limit its therapeutic window and suggest its use should be restricted in surgical and postoperative scenarios. This supports the observed cytotoxicity profile of CHG and Alexidine in our study. A recent 2024 in vitro study comparing CHX with a lactoferrin‑based mouthwash confirmed that CHX significantly reduces cell proliferation in gingival fibroblasts (eightfold reduction) and increases apoptosis and necrosis rates by 25% in HGF‑1 cells, while also impairing wound closure capacity, consistent with the clinically observed side effects of CHX (Saberikia et al. 2025). In contrast, Proguanil exhibited minimal cytotoxicity, suggesting a highly favorable biocompatibility profile that warrants further investigation for applications where tissue tolerance is paramount, despite its limited antimicrobial activity. Similarly, while Amoxicillin demonstrated low cytotoxicity, this potential advantage is negated by its propensity to stimulate biofilm growth at sub‐inhibitory concentrations.

Taken together, these results highlight the multifaceted challenges in managing S. mutans infections. Methodologically, our work demonstrates that combining standard susceptibility tests with sub‑MIC biofilm induction assays and cytotoxicity screening provides a more holistic risk‑benefit assessment than any single endpoint alone—a framework that should be adopted in future antiseptic and antibiotic development pipelines. Placing our findings in the broader context of the 2025 review by Cordisco and colleagues on moonlighting antibiotics (Cordisco and Serra 2025), it is increasingly evident that antibiotics have dual roles beyond growth inhibition: at sub‑lethal doses they function as signaling molecules that modulate bacterial behavior, including biofilm formation. Our results specifically extend this concept to oral bisbiguanides and biguanide‑related compounds in *S. mutans*. The distinct advantages of biguanide compounds lie in their dual antimicrobial and anti‐biofilm capabilities, although their cytotoxicity profiles warrant careful use. The combination of efficacy against clinical isolates and the ability to suppress key virulence genes (*gtfD*, *brpA*) makes them formidable agents, but their safety profile must be improved. The limited efficacy and biofilm‑promoting potential of antibiotics like amoxicillin raise serious concerns.

Future research should explore structurally diverse analogs that maximize efficacy and minimize toxicity. Additionally, strategic combination therapies informed by FIC analyzes may optimize outcomes. This comprehensive evaluation emphasizes the importance of integrating chemical, microbiological, and molecular data to guide the development of novel, effective therapies against *S. mutans* and its biofilms.

## 5 Conclusion

In conclusion, chlorhexidine and its analogs, particularly alexidine, demonstrate strong antimicrobial and antibiofilm activities against *S. mutans*, effectively suppressing key biofilm‐associated gene expression. However, challenges such as cytotoxicity and emerging resistance in clinical isolates highlight the need for careful therapeutic application. The study underscores the potential of targeted combination therapies and the importance of exploring alternative compounds to develop safer, more effective strategies for managing dental caries and biofilm‐related infections.

## Author Contributions

**Hamideh Kamalloo:** methodology. **Mohammadreza Moeininejad:** methodology. **Maryam kiani golshoui:** methodology. **Fatemeh Samadyar:** formal analysis. **Ali Shivaee:** writing – original draft, writing – review and editing. **Behrooz Sadeghi Kalani:** supervision, conceptualization, project administration.

## Ethics Statement

The authors have nothing to report.

## Conflicts of Interest

The authors declare no conflicts of interest.

## Generative Artificial Intelligence (AI)

The idea, methodology, results, and all figures, charts, and tables presented in this article are original. GPT‐4o was used only to edit the handwritten language to make the text smoother.

## Supporting information


Supporting File

## Data Availability

The data that supports the findings of this study are available in the Supporting Information of this article.
