# Supplementary material for: Comparative Analysis of Chlorhexidine Derivatives and Alternative Agents on Streptococcus mutans Viability, Biofilm Formation, and Gene Expression
Source: Microbiologyopen. 2026 Jul 14;15(4):e70360. doi: 10.1002/mbo3.70360 (PMC13369289; doi:10.1002/mbo3.70360)
Supplement: Supplementary file 1 — Supporting File [file MBO3-15-e70360-s001.docx]

| Reference | TM (°C) | Amplicon size (bp) | Sequence | Gene |
| --- | --- | --- | --- | --- |
| The present study | 60 | 160 | F:TGCAAGCGACGGAAAACAAG  R: GCCTGTCAGAGCTTCACCAT | *gtf*D |
| The present study | 60 | 162 | F: AAGCGTCAACGTGAGGTCAT  R: AGCATCGCTGTACCCCAAAA | *brp*A |
| The present study | 60 | 102 | F: TATTCCGACTGCAGGCCTTC  R: GTACGACAGCCAAAAGGGGA | *LuxS* |
| The present study | 60 | 122 | F: GACGCGGCGTGATGAATATG  R: TTTCCTGTTCGGTCGCATCA | *vic*K |
| The present study | 60 | 177 | F: GCTGCATCACCTTGTTTGGG  R: ACGGTTTCTACAGCTGGTCG | *gyrB* |

Characterization of specific primers for target genes in this study.
